# Supplementary figures and images for: SntB triggers the antioxidant pathways to regulate development and aflatoxin biosynthesis in Aspergillus flavus
Source: eLife. 2024 Nov 5;13:RP94743. doi: 10.7554/eLife.94743 (PMC11537487; doi:10.7554/eLife.94743)

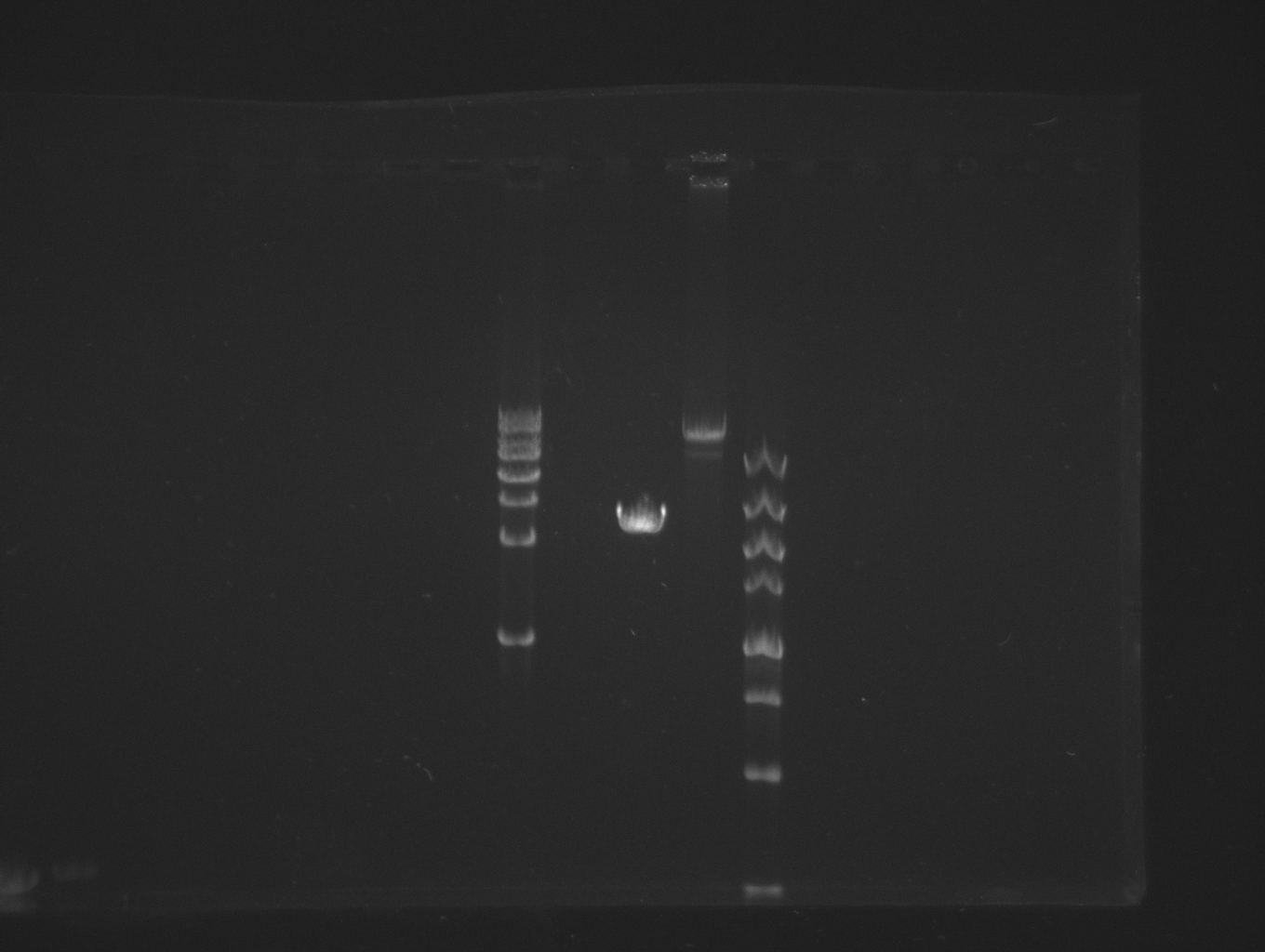

Supplement: Figure 1—figure supplement 1—source data 1. [file elife-94743-fig1-figsupp1-data1.zip › Figure 1-figure supplement 1_Source data 1/snt2-AP.Tif]

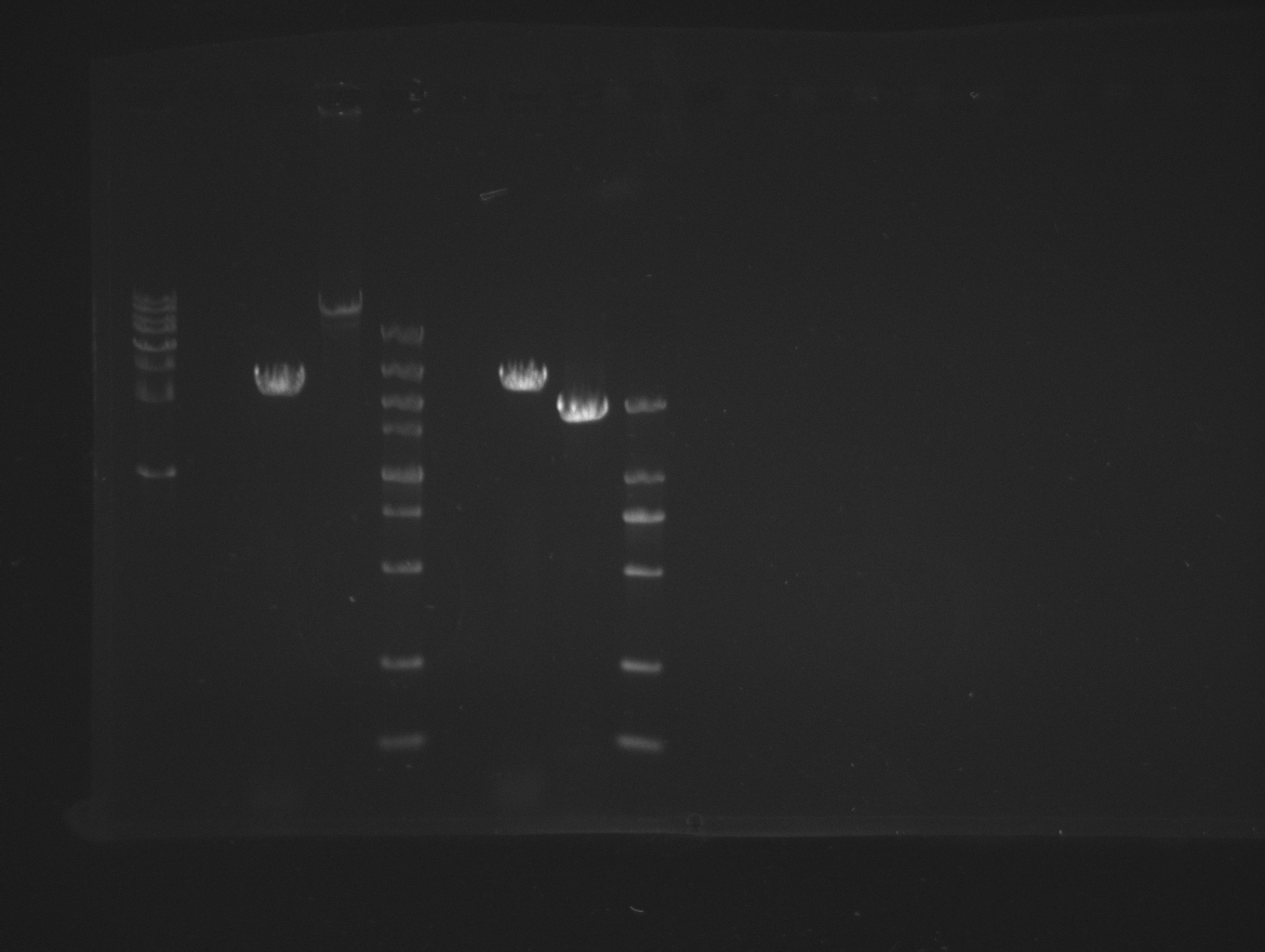

Supplement: Figure 1—figure supplement 1—source data 1. [file elife-94743-fig1-figsupp1-data1.zip › Figure 1-figure supplement 1_Source data 1/snt2-BP.Tif]

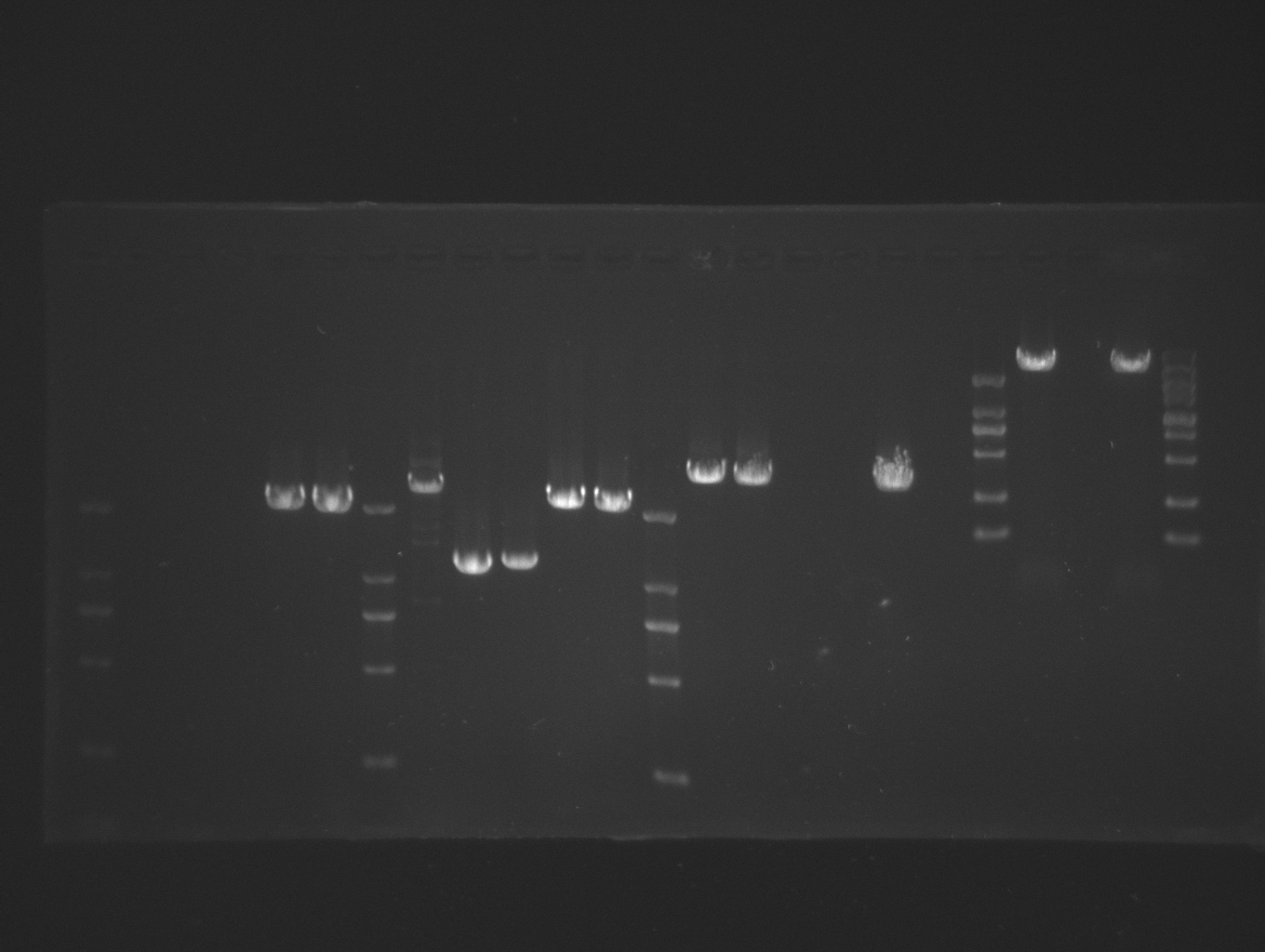

Supplement: Figure 1—figure supplement 1—source data 1. [file elife-94743-fig1-figsupp1-data1.zip › Figure 1-figure supplement 1_Source data 1/snt2-ORF.Tif]

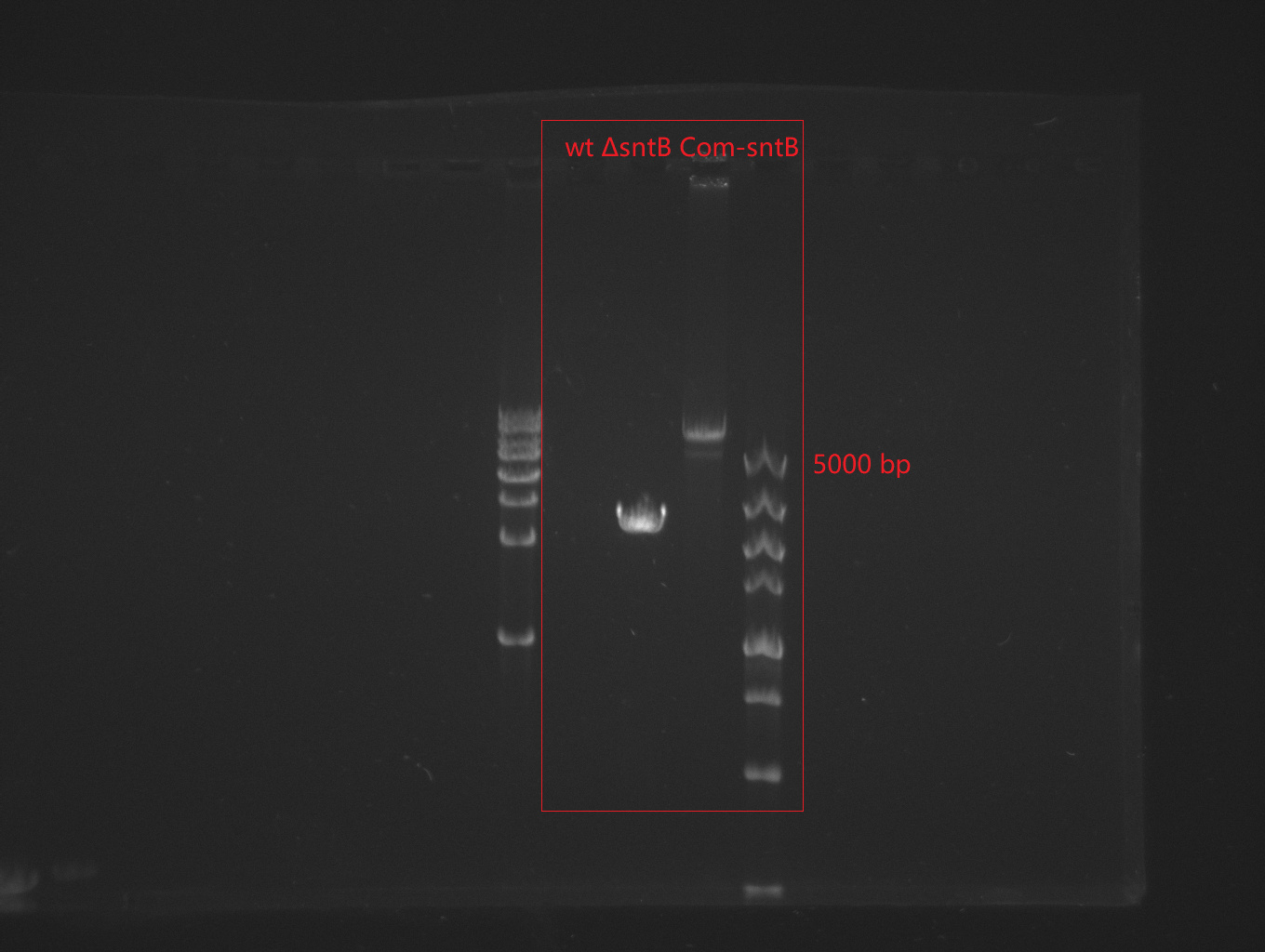

Supplement: Figure 1—figure supplement 1—source data 2. [file elife-94743-fig1-figsupp1-data2.zip › Figure 1-figure supplement 1_Source data 2/snt2-AP1.tif]

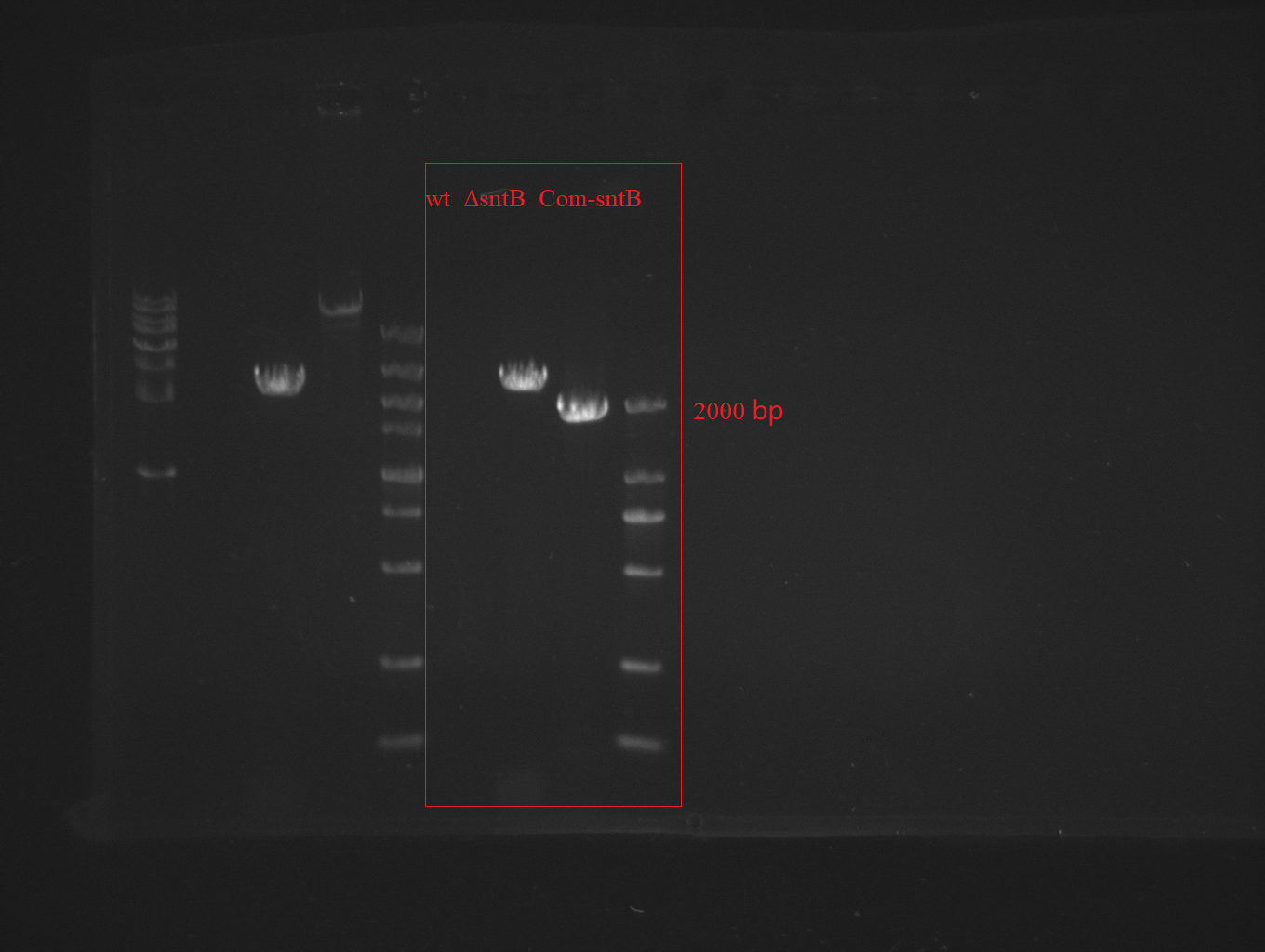

Supplement: Figure 1—figure supplement 1—source data 2. [file elife-94743-fig1-figsupp1-data2.zip › Figure 1-figure supplement 1_Source data 2/snt2-BP1.tif]

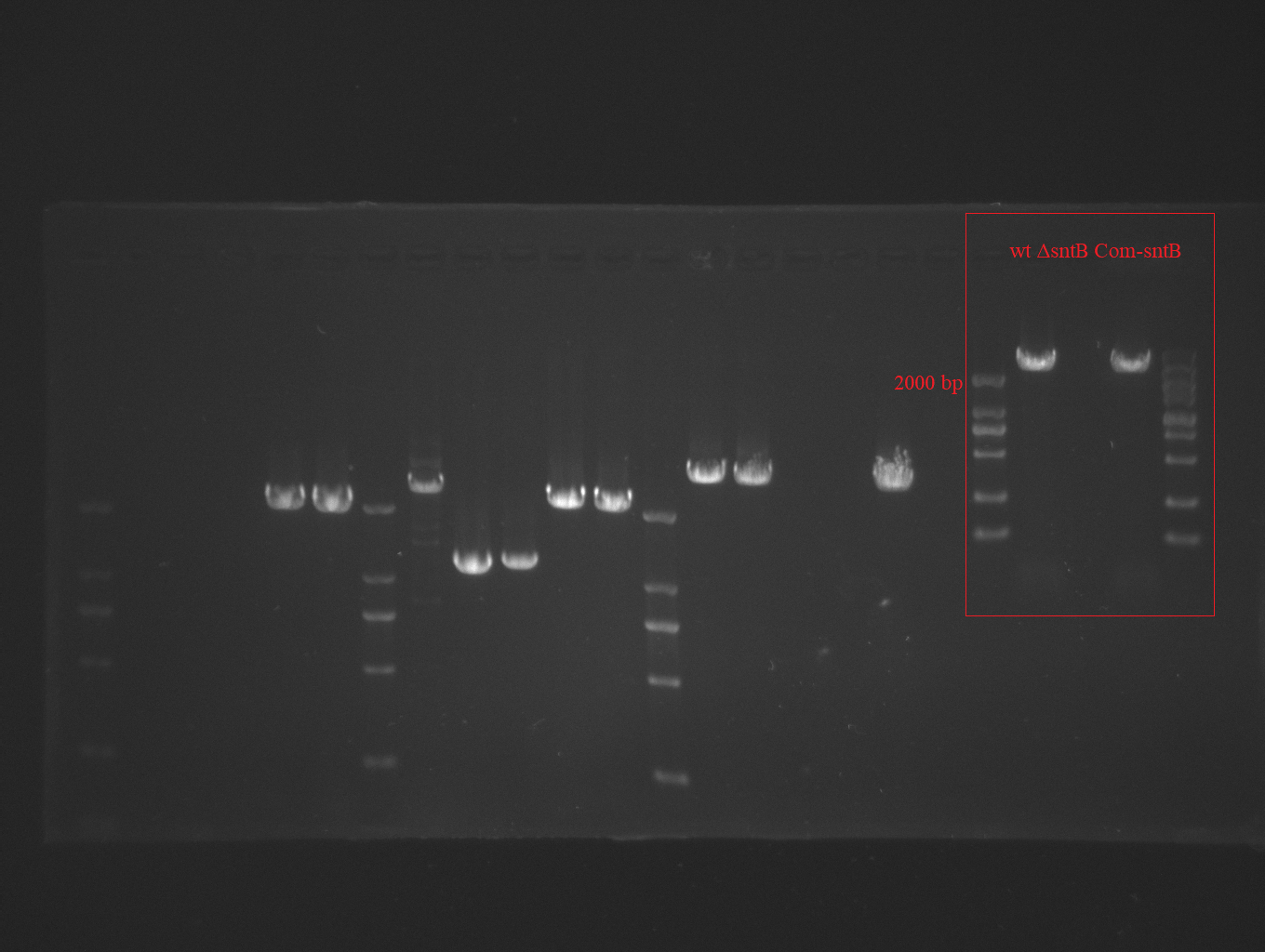

Supplement: Figure 1—figure supplement 1—source data 2. [file elife-94743-fig1-figsupp1-data2.zip › Figure 1-figure supplement 1_Source data 2/snt2-ORF1.tif]

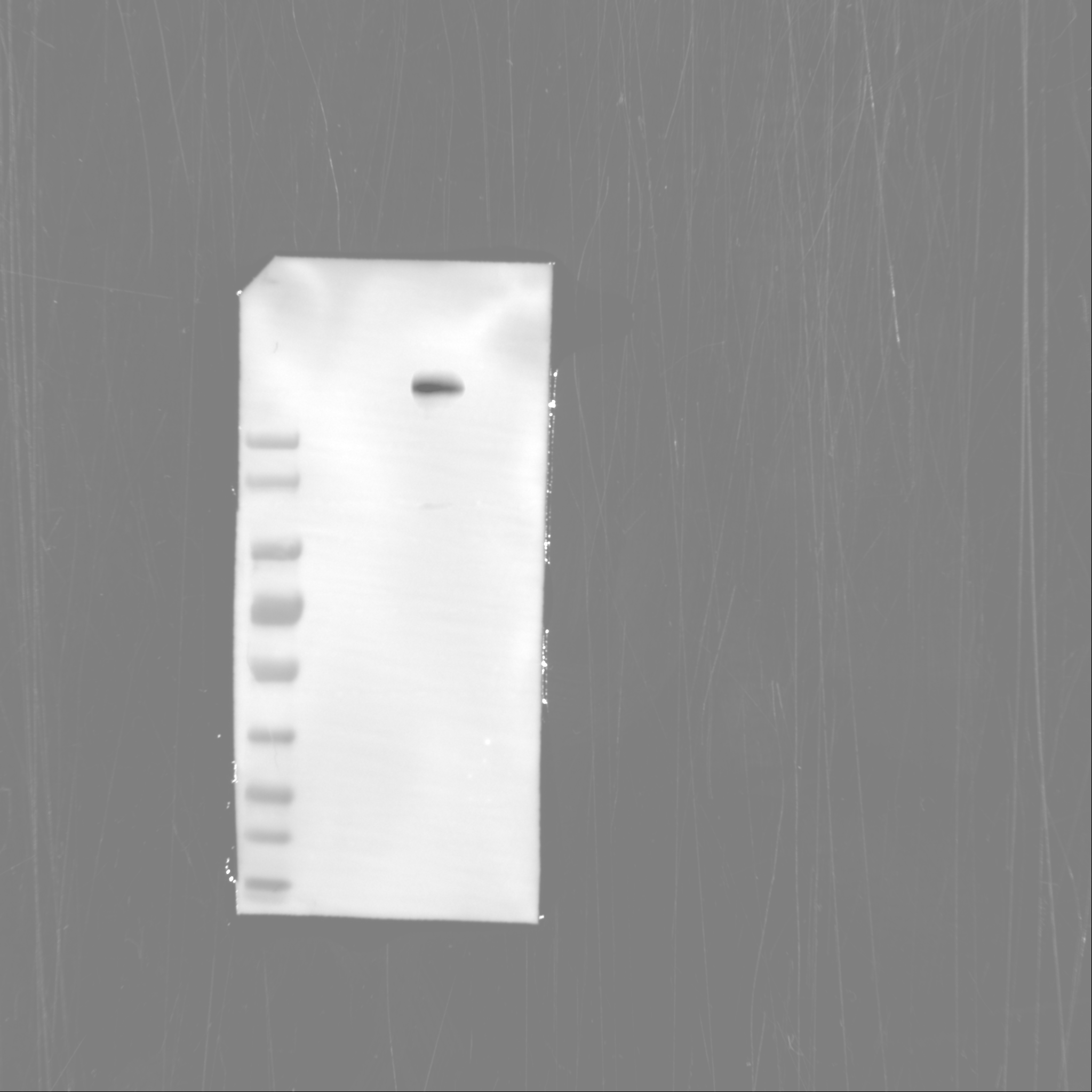

Supplement: Figure 4—source data 1. [file elife-94743-fig4-data1.zip › Figure 4_Source data 1/SNT2-HA.tif]

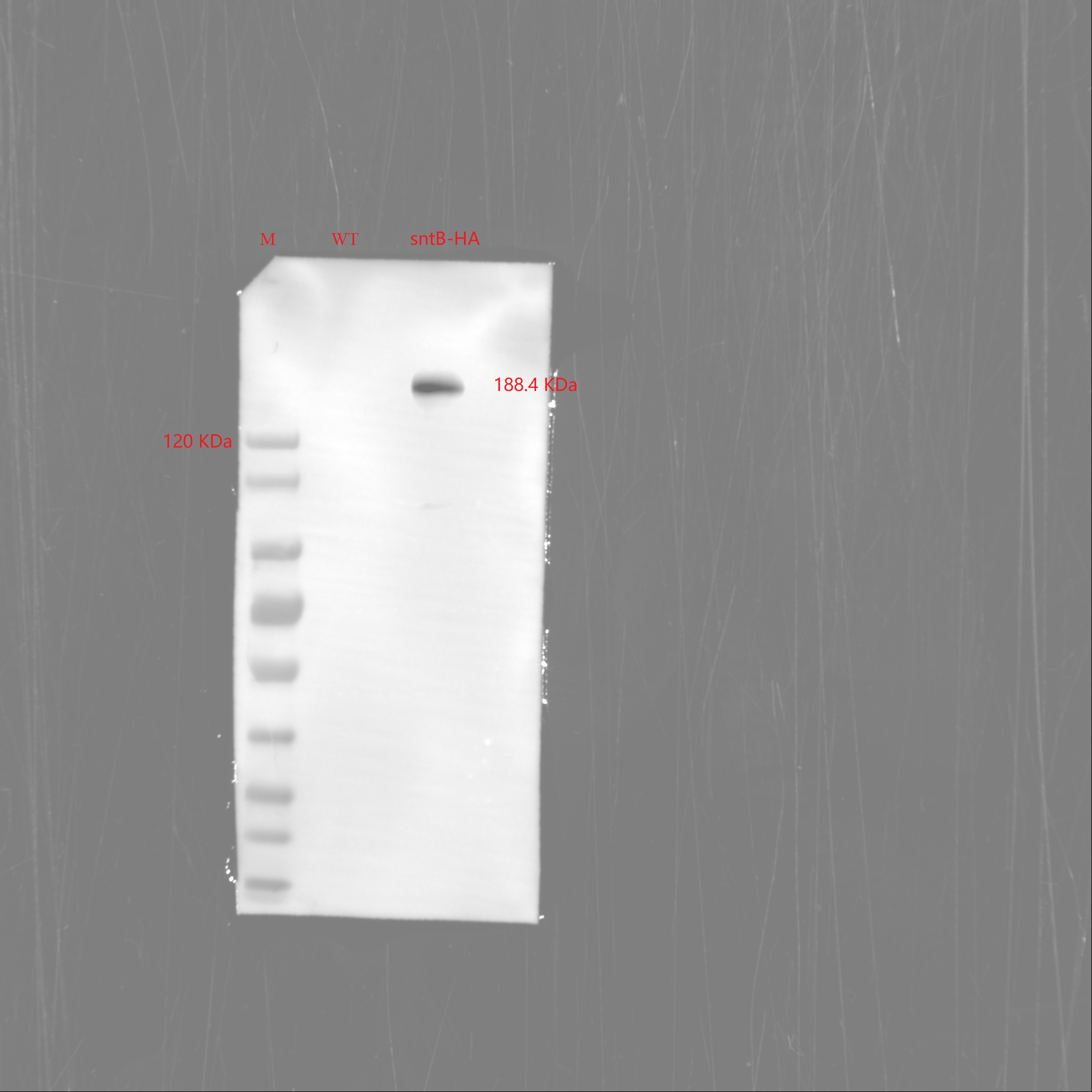

Supplement: Figure 4—source data 2. [file elife-94743-fig4-data2.zip › Figure 4_Source data 2/SNT2-HA.tif]

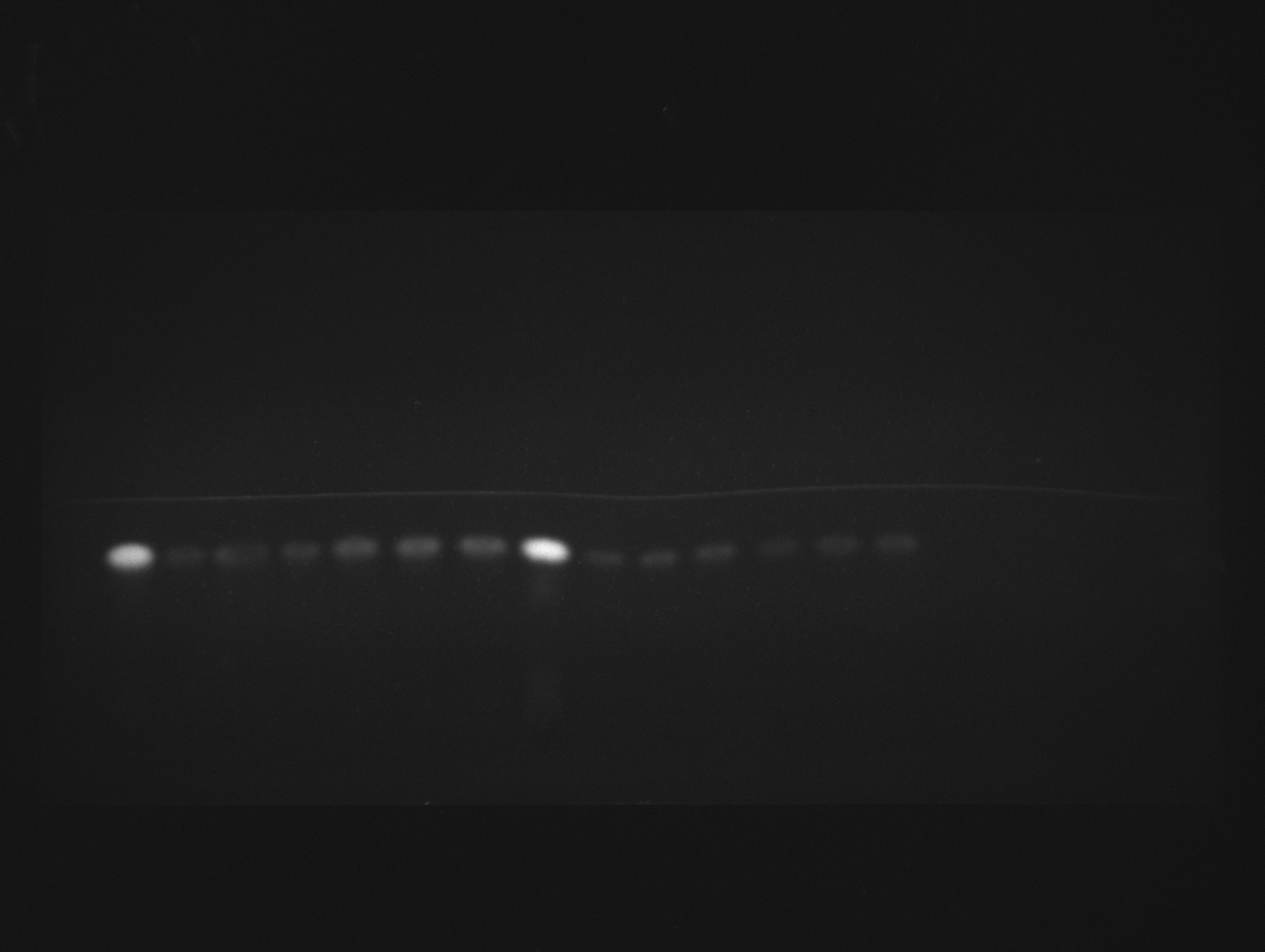

Supplement: Figure 6—source data 1. [file elife-94743-fig6-data1.zip › Figure 6-figure_Source data 1/2023.09.27-WT、catc.Tif]

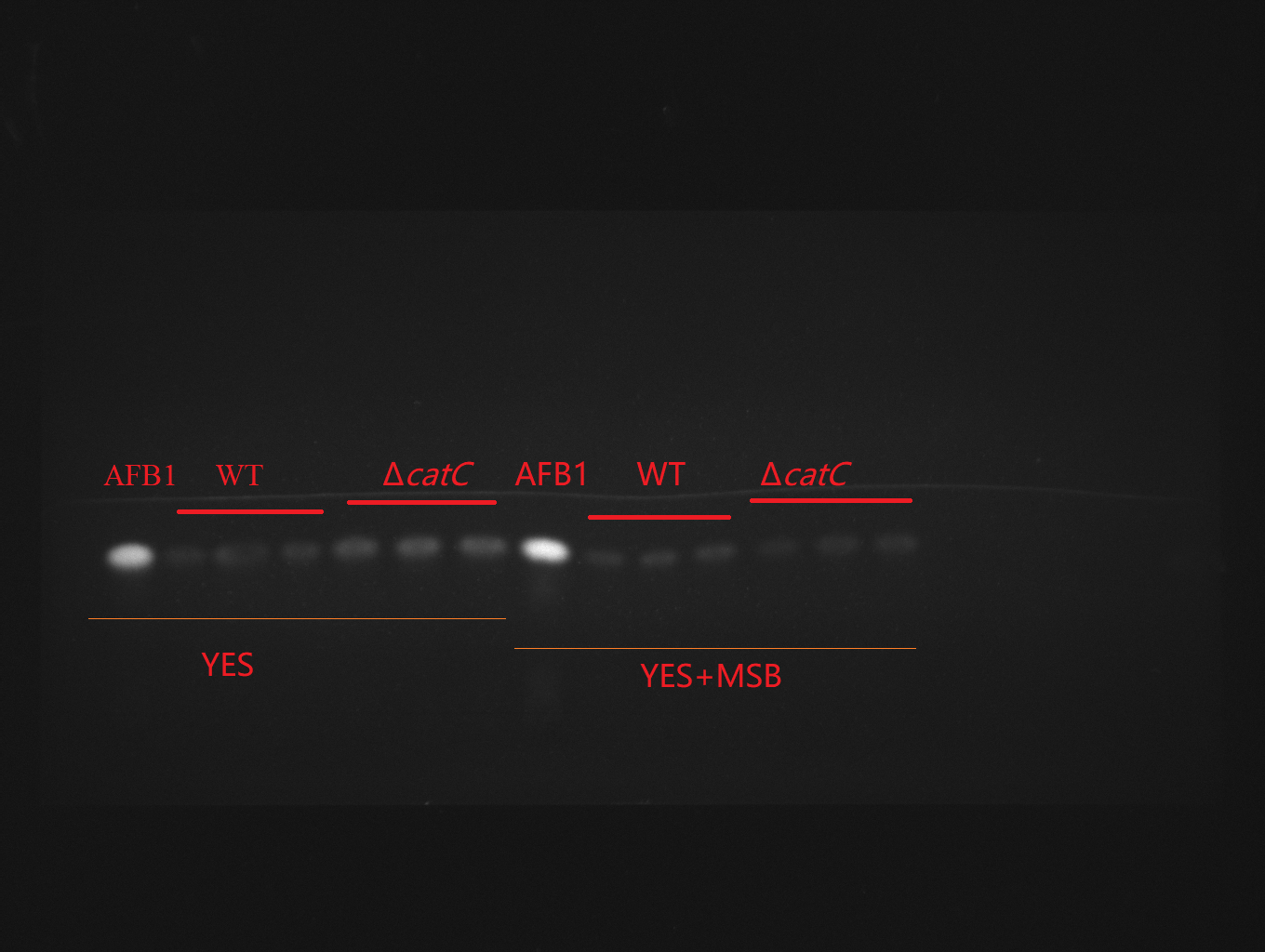

Supplement: Figure 6—source data 2. [file elife-94743-fig6-data2.zip › Figure 6-figure_Source data 2/2023.09.27-WT、catc-withmarker.tif]

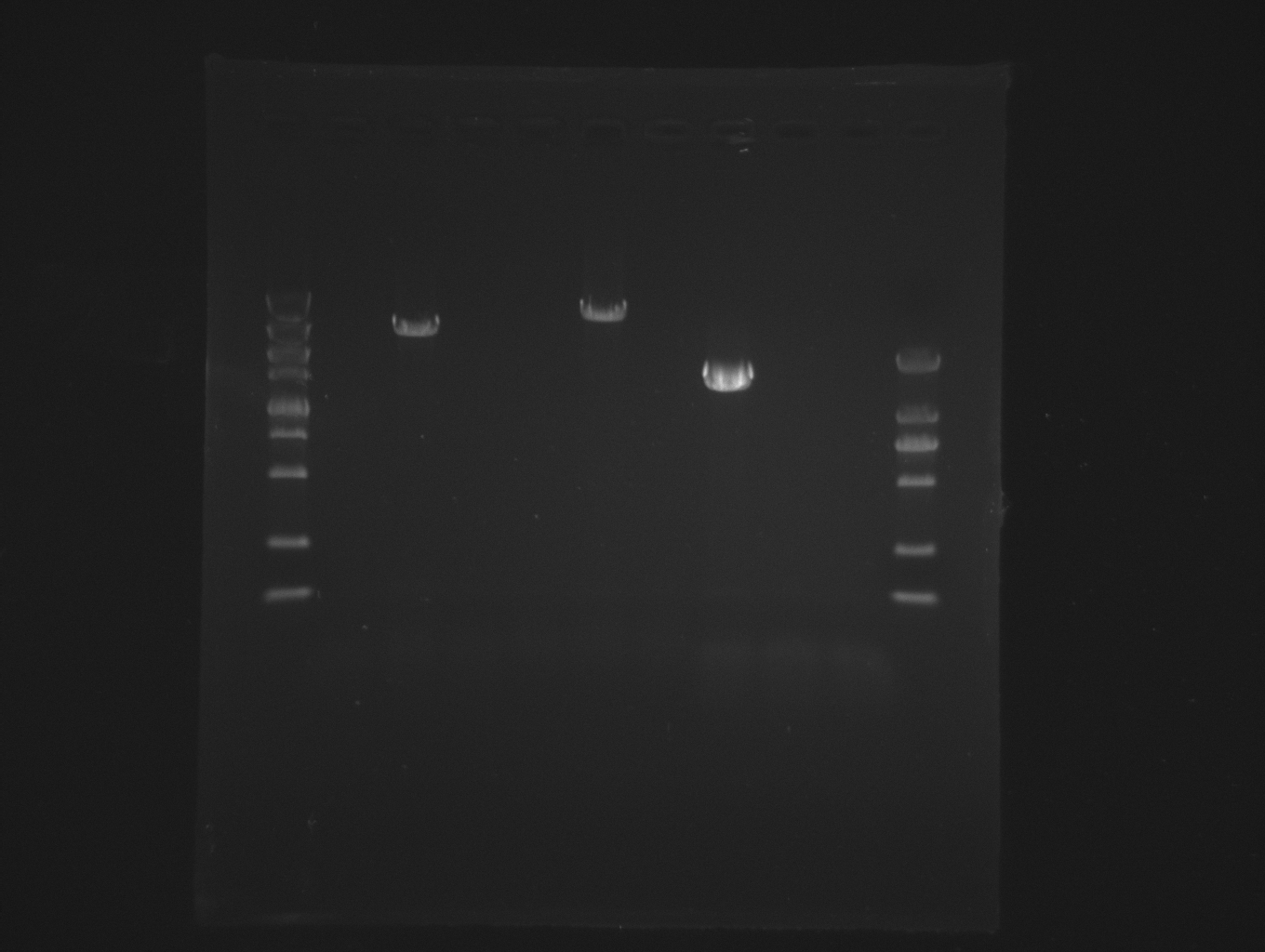

Supplement: Figure 6—figure supplement 1—source data 1. [file elife-94743-fig6-figsupp1-data1.zip › Figure 6-figure supplement 5_Source data 1/catC-AP、BP、ORF.Tif]

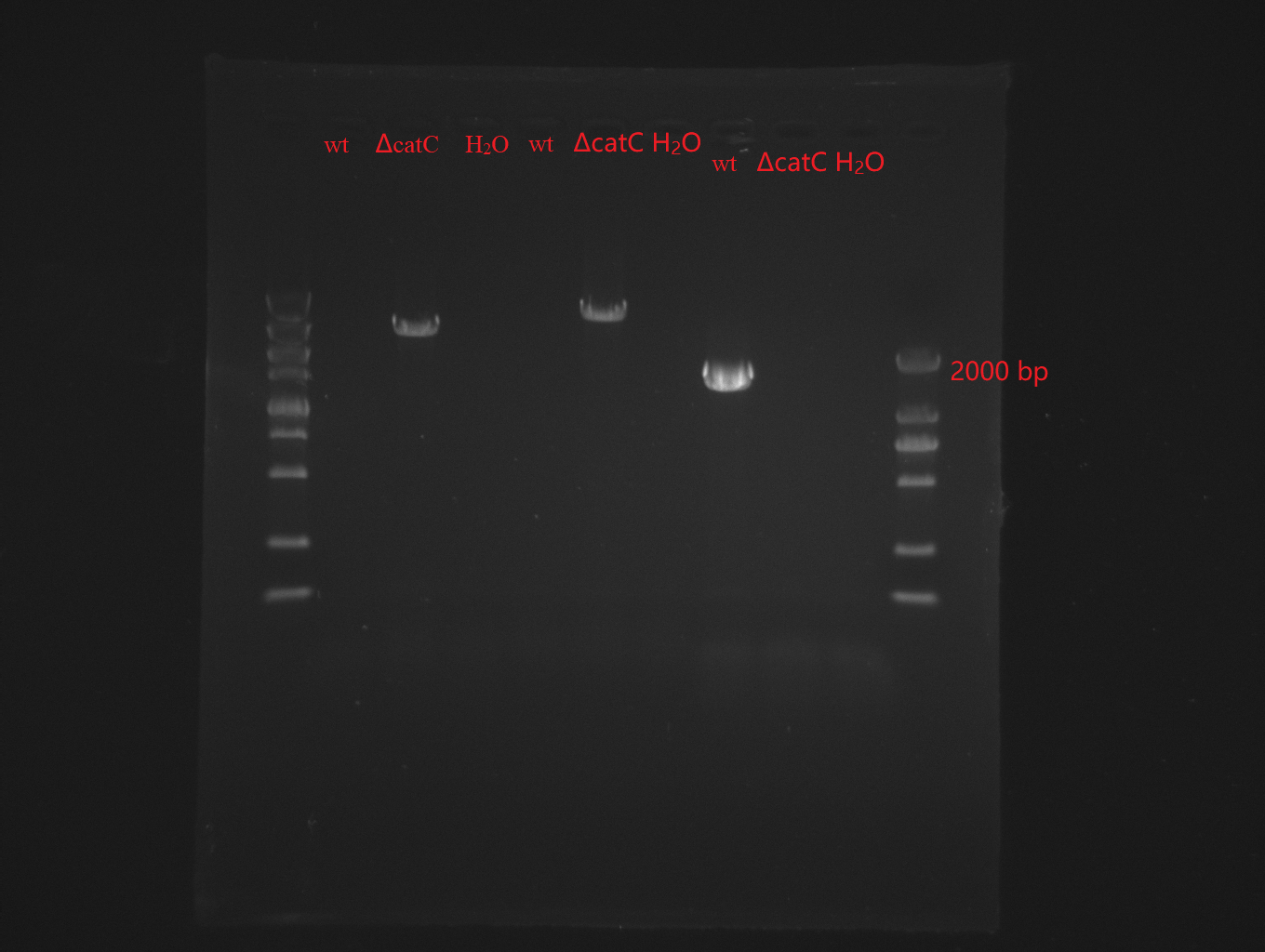

Supplement: Figure 6—figure supplement 1—source data 2. [file elife-94743-fig6-figsupp1-data2.zip › Figure 6-figure supplement 5_Source data 2/catC-AP、BP、ORF.Tif]
